# Dynamic linkages between chicken meat production, consumption, income and trade: Evidence from Wavelet coherence and Granger causality in Asia

**DOI:** 10.1016/j.psj.2026.106733

**Published:** 2026-03-06

**Authors:** Yasodara Silva, Himaya Susan, Nisal Perera, Kalana Mendis, Ruwan Jayathilaka, Umesha Dabare

**Affiliations:** a SLIIT Business School, Sri Lanka Institute of Information Technology, New Kandy Road, Malabe, Sri Lanka; b Department of Information Management, SLIIT Business School, Sri Lanka Institute of Information Technology, New Kandy Road, Malabe, Sri Lanka; c Department of Business Management, SLIIT Business School, Sri Lanka Institute of Information Technology, New Kandy Road, Malabe, Sri Lanka

**Keywords:** Chicken meat production, Chicken meat consumption, Gross domestic product, Granger causality analysis, Trade openness

## Abstract

The poultry industry has become one of the fastest-growing agricultural sectors in Asia, driven by rising incomes, and shifting food preferences. Therefore, this study aims to examine the relationship between chicken meat production and key determinants, including chicken meat consumption, gross domestic product, and trade openness, over 30 years (1993-2022) across 28 Asian countries. This study's foundation was based on the theories of consumer demand and international trade. Wavelet coherence and Granger causality analysis were utilised to identify the direction of causality of the variables. The Wavelet results reveal that chicken consumption and GDP become most significant with the production in the Asian continent, while Granger results reveal that most Asian countries showed unidirectional causal flows from trade openness to chicken meat production and from chicken meat production to gross domestic product and consumption. Furthermore, this study provides novel insights that inform policy considerations for policymakers, international and domestic organisations, and governments, aligning with the Sustainable Development Goals established by the United Nations.

## Introduction

In the past few decades, global meat consumption has increased mainly due to population growth, urbanisation, and income growth. Conversely, the reasons for significant increases in livestock husbandry (Ahmadi Kaliji et al., 2025; Sammani et al., 2025; Steinfeld et al., 2006). Consumers consider meat an essential source of protein, with chicken being the preferred choice, as noted by (Cisternas et al., 2024). According to the Food and Agriculture Organisation, the poultry industry is one of the fastest-growing livestock sectors globally (Shioda et al., 2024), with 50% of poultry meat expected to increase in quality and affordability by 2025 (Mahmood et al., 2025; Silva et al., 2026). Moreover, the chicken population is expected to grow to 37 billion, and as a result, concerns about food security have also risen in livestock production (Kleyn and Ciacciariello, 2021; Tasdelen and Arslan, 2025), due to the high consumption.

Global chicken meat production (CMP) has reached unprecedented levels. The Asian continent stands out as one of the most prominent producers and consumers in the poultry industry (Mahanty et al., 2023). In 2023, it accounted for 58.09 million tons of production, surpassing other continents since 1997. (Food and Agriculture Organisation, 2025c) making Asia a large contributor to the poultry production.

Consuming poultry meat is nutritious and a food security measure for most countries in Southeast Asia, including Indonesia, Laos, the Philippines, Thailand, and Vietnam (Soon-Sinclair et al., 2024). Furthermore, in India, poultry production is also a rapidly growing sector within the livestock industry, driven by economic development, population growth (Mehta and Nambiar, 2007), and urbanisation (Devendra, 2007; Sequeira et al., 2025). Furthermore, regarding pricing preferences, most consumers in Asia favour chicken meat (Cisternas et al., 2024). Therefore, this study solely contributes to countries in the Asian continent.

**Fig. 1:**
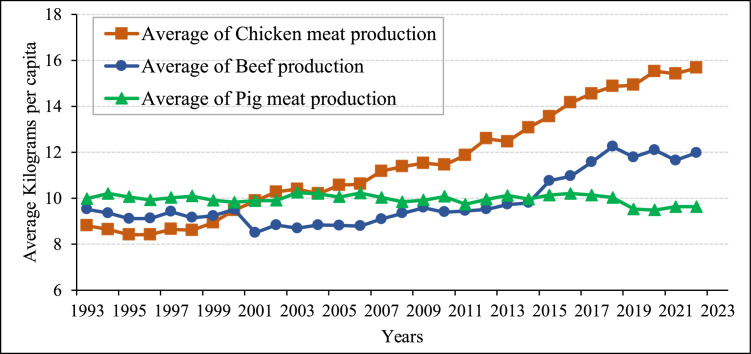
Trend pattern of meat types for the Asian continent.

Source: Authors’ illustration based on the Food and Agriculture Organisation (2025a).

The trend patterns of production in three meat types for the Asian continent, as shown in Fig. 1, indicate that chicken meat production has increased significantly compared to beef and pig meat production. Therefore, in this study, chicken meat production has been the focus for the Asian continent to enhance the sustainability of the poultry industry.

### Critical literature review

The meat industry plays a significant role in the world of agriculture and food, and the poultry sector is considered to be the largest sector of agriculture (Choudhury et al., 2025). It continues to expand with consistent demand and sustainable food production. Worldwide, chicken meat is one of the most widely consumed meat types (Wang et al., 2023), and the most consumed poultry meat type (Matsuda et al., 2025; Ning et al., 2025; Qi et al., 2025). In the future, it is expected to lead global meat production (Food and Agriculture Organisation, 2024).

One of the major chicken meat consumers and producers is considered to be the Asian region (Akhtar et al., 2023; Li, 2025; Mahanty et al., 2023), where China accounted for producing 15.35 million chicken meat as of 2024 (United States Department of Agriculture, 2025b), taking the lead in Asia but also focusing more on food security (Zhu, 2016) in the domestic market.

Asia accounted for 38% of the world's chicken meat production in 2020 (Food and Agriculture Organisation, 2025b). Chicken meat is primarily valued for its high nutritional value (Mishra et al., 2023), as a valuable source of protein (Dapanas and Niepes, 2024; Sparrow et al., 2024), and the affordability of the meat (Abeyesinghe et al., 2025; Debbarma et al., 2024; Gkarane et al., 2020). Furthermore, numerous studies suggest that improving the quality of chicken meat requires specific dietary interventions.

In Southeast and East Asia, there is a high value placed on Japanese native chickens. Additionally, due to its high quality, indigenous chicken meat is highly preferred across most countries in Asia (Chowdhury et al., 2014; Ranasinghe et al., 2024). The yellow-feathered boilers, a special breed in Asia, can contribute to reducing food waste (Zhang et al., 2016). They are consumed more than live chickens due to disease outbreaks in Asia (Wang et al., 2017), and these outbreaks pose a greater threat to the entire poultry industry (Nguyen et al., 2024). Poultry farming in rainfall-affected areas of South Asia is expected to be more profitable, enabling the production of high-quality chickens (Bhagat and Dwivedi, 2024). Additionally, in Malaysia, due to high demand, there is a need for sustainable chicken production (Samsuddin et al., 2015). This suggests a significant relationship between chicken production and consumption in Asia that warrants further scrutiny.

The poultry industry significantly contributes to the country's GDP, as poultry production is a reliable source of income (Gilbert et al., 2015; Idowu et al., 2021; Whitton et al., 2021) for upper-middle-income, lower-middle-income, and low-income countries (Shioda et al., 2024). Moreover, with the rise of poultry production, global income levels have also increased (Khan et al., 2021). Poultry production has contributed to improving the GDP by increasing its output by 135%, primarily due to substantial investments in large-scale poultry farms. Antimicrobials help maintain low costs in poultry production, enhancing high income (Coyne et al., 2019). The Backyard chicken system is one of the most common production methods in low and middle-income countries (Chauhan et al., 2024; Ibrahim et al., 2025; Muñoz-Gómez et al., 2025; Muñoz-Gómez and Torgerson, 2024), and practised by 80% of the global rural population (Conan et al., 2012). COVID-19 reduced the trade market and negatively affected economic growth in developing nations (Attia et al., 2022; Das and Samanta, 2021) and the Asian region.

In the context of Asia, the development of the local chicken industry in Indonesia has significantly contributed to the agricultural sector's revenue generation (Sumantri et al., 2020). Furthermore, in low- and upper-middle-income countries such as Vietnam, Chicken flocks are a primary source of income for households (Truong et al., 2021). Income growth has catalysed the development of poultry production and consumption in South Asian states, such as Indonesia, Malaysia, and Thailand (Lee and Hansen, 2019). Additionally, in Bangladesh, Pakistan, Vietnam, and Sri Lanka, people have increasingly focused on indigenous chicken production as their household income has also risen (Bett et al., 2014). Due to high living standards, global chicken production is expected to quadruple by 2050, including in Asia (Attia et al., 2024). Indeed, there seems to be a nexus between CMP and GDP in the Asian region.

The international market has provided the chicken industry with an opportunity to grow exponentially. The high demand for chicken products has led countries to ship billions of chickens to meet global demand (Arp et al., 2024; Radin et al., 2017). The Asian region is considered one of the largest exporters of chicken meat products, mainly due to its high production rate, with China being the most significant contributor (Henry & Rothwell, 1995; Wen et al., 2019). With the rise of chicken imports, China’s GDP was unable to keep pace with the rising demand (Li et al., 2023). This suggests that China should focus more on the domestic market to boost the nation's GDP rate.

Another Asian country and a top 10 poultry-producing and exporting country is Thailand, which contributes significantly to global chicken production. However, regarding chicken imports, Russia faced trade embargos from the US, Canada, Australia, and the EU (Soon and Thompson, 2020), and from 2015 to 2019, local chicken production decreased due to the removal of non-tariff barriers and which led to an increase in imports by 326-423 thousand tons in Russia (Soon and Thompson, 2020). Due to the tariff reduction in Indonesia, imports have increased, which has affected domestic output (Firmansyah et al., 2018). On the other hand, during seasonal periods, an increase in poultry production and consumption was observed in Thailand (Klaharn et al., 2024). There appears to be a link between CMP and TO in the Asian region that warrants examination.

After reviewing past literature, it has revealed that a knowledge gap exists in previous studies, as most studies have utilised only one or more variables in a single country or multiple countries together.

Therefore, this study addresses that knowledge gap by investigating the causal relationship between chicken meat production and three independent variables: chicken meat consumption (CMC), gross domestic product (GDP), and trade openness (TO) for the Asian continent, comprising 28 countries over 30 years. This study makes three significant contributions to the existing literature in an insightful manner. Unlike conventional panel studies that rely solely on time domain estimators, this research integrates time-frequency Wavelet coherence with country-specific Granger causality to capture heterogeneous dynamic interactions across short, medium, and long run horizons with the single field framework.

First, a dual methodological approach has been taken to get the macro picture, which involves Wavelet coherence analysis to find the direction and the coherence of the nature between the variables, and another approach to see the micro picture by utilising the Granger causality analysis to find the causality direction of the variables within the countries to provide a deep perspective.

Second, this study presents attractive visualisations to utilise the analysis results and provides a deeper understanding of the causal flows of the variables, along with country-specific interpretations.

Finally, the results have paved the way for novel findings that provide policy initiators with new perspectives to enhance the sustainability of the chicken meat industry and the agricultural sector, aligning with the United Nations' Sustainable Development Goals (SDGs).

This study addresses specific SDGs by focusing on SDG 1: No Poverty, SDG 2: Zero Hunger, SDG 3: Good Health and Well-being, SDG 8: Decent Work and Economic Growth, SDG 9: Industry, Innovation, and Infrastructure, SDG 12: Responsible Consumption and Production, and SDG 17: Partnerships for the Goals.

### Theoretical perspective

The theories presented here help justify and solidify the survey by providing a theoretical framework for the study.

The consumer demand theory, which explains that consumers will decide what to buy by choosing a combination of goods that provides the most satisfaction (Koutsoyiannis, 1979), while staying within a fixed price and income limits (Basmann, 1956; Böhm & Haller, 2017). And also, the consumer demand for chicken meat will be varied due to its high-quality protein (Fanatico et al., 2025), antibiotic-free boilers (Haque et al., 2020), affordability (Mahmood et al., 2025), being free from religious restrictions (Daghir et al., 2021) and having other nutrients as well.

**Fig. 2:**
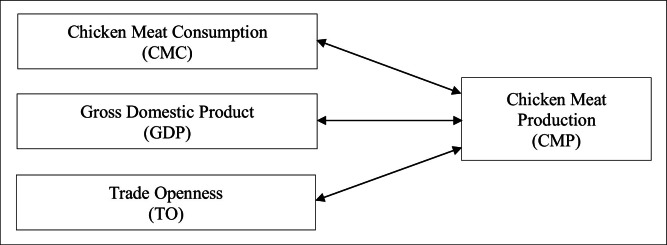
Conceptual model.

Source: Authors’ compilation.

International trade theory is the physical or electronic transmission of goods and services across country boundaries (Ezenwa et al., 2021; McConnell et al., 2001). In the context of chicken meat production, trade openness is an advantage to expand the international trade market. However, trade openness will vary from country to country due to animal diseases (Otte et al., 2008; Rushton et al., 2005), trade barriers, food safety concerns (Jongwanich, 2009), rules and regulations, and climate change (Lin et al., 2022). Therefore, based on the theoretical perspective of the study, a conceptual model has been developed for the variables presented in Fig. 2.

The conceptual model has been drawn considering the CMP as the dependent variable, with the independent variables being CMC, GDP, and TO, to illustrate the interconnectivity of the variables. Although the conceptual framework positions CMP as the dependent variable based on economic production theory, the empirical analysis does not impose a strict unidirectional structure. Instead, the use of Granger causality testing allows the data to reveal potential bidirectional feedback relationships among the variables. This approach is consistent with modern empirical economic modelling, where theoretical expectations guide the model structure while statistical methods evaluate whether real-world dynamics conform to or deviate from those expectations.

The following sections of this paper present the data and methodologies, results, and a discussion of the relevant findings, along with conclusions, policy implications, limitations, and future research possibilities.

## Data and methodology

This section provides details on the data and statistical methodologies used to analyse those variables and obtain the results in this study.

### Country and variable section criteria

In this study, countries were included based on three criteria. First, the continuous availability of data for all variables from 1993 to 2022. Second, consistency of reporting across FAOSTAT and World Bank databases. Third, the absence of structural data breaks exceeding three consecutive years.

Countries with missing series or inconsistent measurement units were excluded to ensure panel balance and comparability. The full panel dataset is presented in Appendix 1, while the variable definition and the sources are summarised in Table 1. Variables were selected based on theoretical relevance to the production system and international trade dynamics, consistent with consumer demand and the trade theory framework.

**Table 1:** Data sources and variables.

**Variable**	**Measurement**	**Source**
Chicken Meat Production (CMP)	Kg per capita	(Food and Agriculture Organisation Statistics, 2025a)
Chicken Meat Consumption (CMC)	Kg per capita	(Food and Agriculture Organisation Statistics, 2025b)
Gross Domestic Product (GDP)	Per Capita (current US$)	(World Bank, 2025a)
Trade Openness (TO)	Percentage of GDP	(World Bank, 2025b)

Source: Authors’ compilation.

### Methodology

**Fig. 3:**
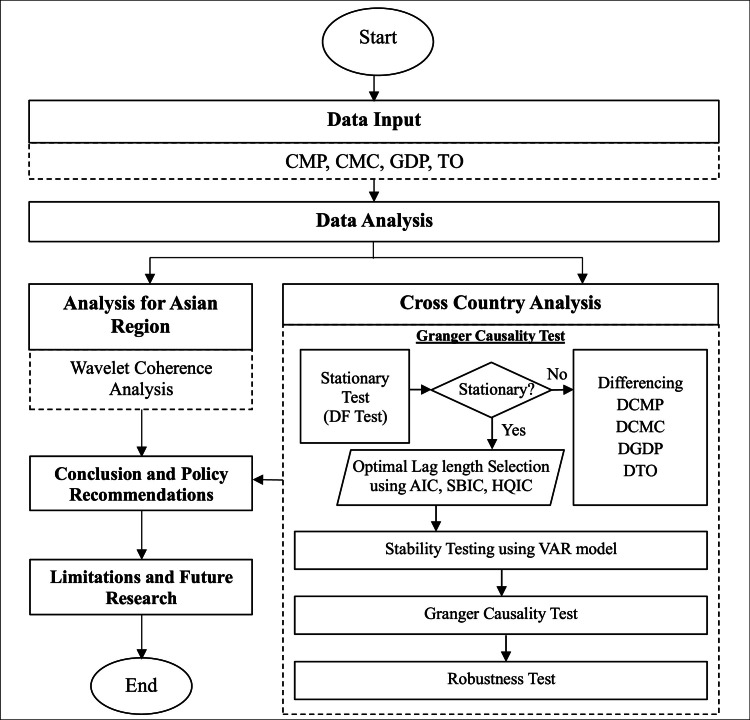
Methodology flow diagram.

Source: Authors’ illustration.

The methodologies employed in this study were used to explore the nexus between the variables through Wavelet coherence and Granger causality analysis. The steps and procedures used for the Wavelet coherence and Granger causality analysis were showcased in Fig. 3.

The Wavelet coherence methodology was beneficial for exploring coherence among variables with different frequencies across different terms, and Granger causality analysis helped gain a deeper understanding of the direction of causality across Asian countries.

Although panel econometric models, such as panel VAR or panel cointegration, are commonly used for cross-country datasets, they impose a homogeneity assumption across units and primarily estimate average relationships. The objective of this study, however, is to uncover country-specific causal dynamics rather than pooled effects. Because Asian economies differ substantially in institutional structures, production systems, trade regimes, and consumption behaviour, panel estimators may fail to capture meaningful heterogeneity.

Therefore, country level granger causality analysis was employed to preserve structural differences and identify distinct causal pathways for each country, which is more consistent with the study's comparative analytical objective.

#### Wavelet coherence analysis

When analysing time series data, the Wavelet coherence methodology is an advanced analytical tool (Banović et al., 2013; Caldera et al., 2024; Wijesuriya et al., 2025) that can analyse the nature of the coherence and the direction of the causal relationships in different time frames at a frequency level, which can provide valuable insight into the variables.

This methodology has been employed in past studies spanning various sectors (Jayathilaka et al., 2025; Sehgal and Singh, 2025), which encompasses multiple fields.

(1) $$\psi^{a,b}\left(x\right)={\left|a\right|}^{-\frac{1}{2}}\psi \left(\frac{x-b}{a}\right)$$

The statistical expression for the Wavelet coherence methodology is shown in Eq. 1, where the scale and translation parameters are denoted as a and b, respectively. The Morlet Wavelet function is represented in $\psi^{a,b}$.

Wavelet methodology helps identify the nexus between chicken meat production and independent variables at the frequency level, as well as the nature of coherence. Additionally, provide advantages that reveal how each variable influences in different terms of the period. Graphical illustrations are created using the R software, which allows for convenient interpretation.

#### Granger causality analysis

Granger causality analysis is an advanced, sophisticated methodology that has been employed in this study to identify the direction of causality at a country level, and this methodology has been utilised in various studies (Athalage et al., 2025; Galappaththi et al., 2023; Palliyaguru et al., 2024; Wijesuriya et al., 2025), which are relevant to diverse sectors. Before conducting analysis, the Dickey-Fuller (DF) unit root test (Dickey and Fuller, 1979) is useful for checking stationarity in a time series dataset. When finding the existence of a unit root in a variable, it should convert to differences until that variable is stationary. After that, using the Vector Autoregression (VAR) model by conducting Akaike’s Information Criterion (AIC) (Akaike, 1998), Schwarz’s Bayesian Criterion (SBIC) (Schwarz, 1978) and the Hannan and Quinn Information Criterion (HQIC) (Hannan and Quinn, 1979) find the minimum criteria as optimal lag length and check the stability of the model accordingly. Once these prerequisite tests are completed, we proceed to the Granger causality analysis and its statistical expression, as shown in Eq 2.

(2) $$\;Y_{i,t}=\sum_{k=1}^{\rho }\beta_{i}Y_{i,t-k}+\sum_{k=0}^{\rho }\pi_{k}X_{i,t-k}+u_{i,t}$$

Independent and dependent variables are represented by X and Y, respectively, while i and *t* denote the country and the period, respectively. Number of lags symbolised by ρ and frequency of lags shown by *k*. The coefficient of the regression indicates in β and π are constant value of the regression, where k may take any value within the range of population size. Error term depicted in u.

Afterwards, to confirm the reliability of the Granger results, it is helpful to employ robustness checks by utilising different lag lengths.

This methodology helps explore the causal relationship within countries, while being beneficial for identifying the direction of causality in each country in response to specific environmental changes. Additionally, suitability for employing time series data to forecast future values. This study employs it on a data series for Asian countries across three decades.

Compared with conventional panel estimators that assume parameter stability over time, the Wavelet coherence and Granger framework allows relationships to vary across both time and frequency domains. This is particularly important in agriculture production system where structural shocks, disease outbreaks, trade disruptions, and demand fluctuations generate non-stationary and heterogeneous dynamics. Therefore, a selected methodologies intentionally designed to capture the multi scale casual patterns rather than average effects.

It is important to note that Granger causality reflects predictive relationships rather than structural causations. Accordingly, the results should be interpreted as indicators of dynamic interdependency rather than definitive causal mechanisms.

## Results and discussion

This section presents the findings derived from descriptive statistics, Wavelet coherence analysis, and Granger causality analysis, along with a comprehensive discussion of the results.

### Descriptive analysis

**Fig. 4:**
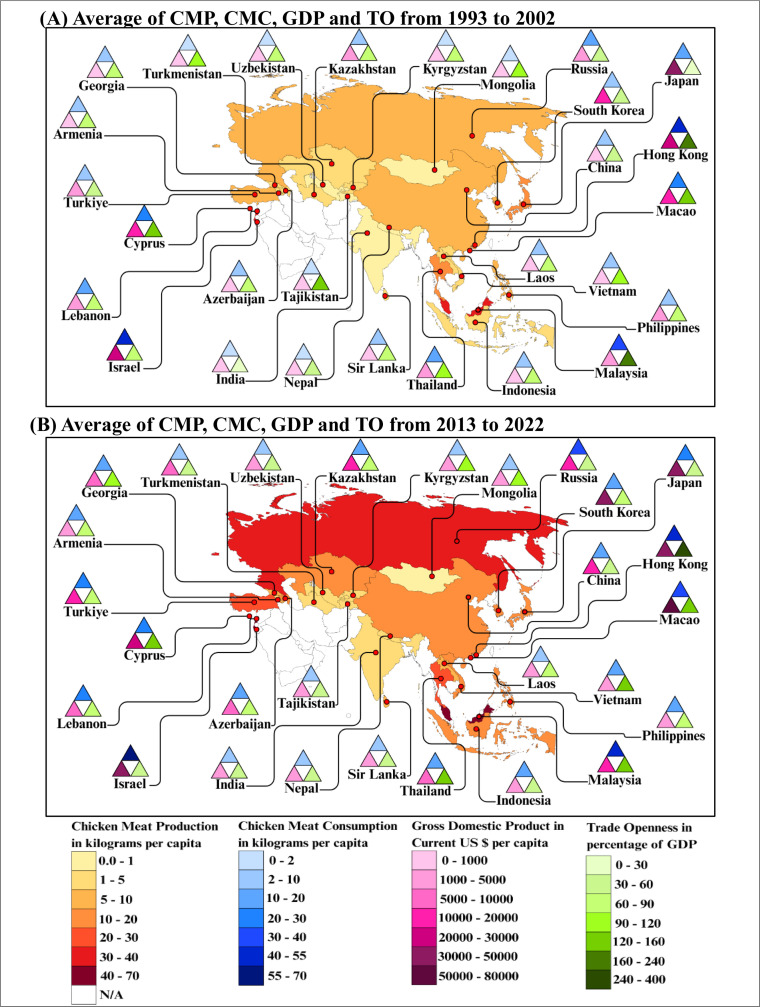
Average of chicken meat production, chicken meat consumption, GDP, and trade openness for two periods from 1993 to 2002 and 2013 to 2022. Note: The triangle shapes in the map chart represent the independent variables, with blue, pink, and green colours representing chicken meat consumption, GDP, and trade openness, respectively. The middle white colour triangle does not represent any independent variable. The map visually compares how these variables vary across the Asian continent over different time periods.

Source: Authors’ illustration based on the Food and Agriculture Organisation (2025a) and World Bank (2025a).

In this study, descriptive statistics were used to get a basic understanding of the variables. Appendix 2 presents a summary of descriptive statistics for the variables, and Fig. 4 displays the averages of CMP, CMC, GDP, and TO for two time periods: 1993-2002 and 2013-2022.

When compared to Fig. 4 (A), Fig. 4 (B) shows an increase in gross domestic product for every country in Asia. Furthermore, during this period, there has been a significant rise in CMP and CMC in countries such as Georgia, Israel, Kazakhstan, Malaysia, Russia, South Korea, and Turkey. At the same time, a decrease is observed in countries such as Hong Kong and Cyprus. In addition, the highest trade openness is in Hong Kong, Cyprus, Macao, and Malaysia, while the lowest trade openness is in India, China, Nepal, and Sri Lanka.

### Wavelet coherence analysis

The Wavelet coherence graphs express the correlation between the variables by considering the grey-shaped cone for the analysis. The black lines represent the 5% significance level, as determined by the Monte Carlo Simulation. Then, the area in red/orange represents the existence of a correlation, and the area in blue represents the non-existence of a correlation in the graph. The scale range is set to 0 to 256, with intervals from 0 to 16, 16 to 64, and 64 to 256 considered short-term, medium-term, and long-term, respectively.

The guidelines for additional interpretation are provided in Table 2 to get a wider understanding of the Wavelet coherence graphs.

**Table 2:** Interpretation of wavelet coherence.

Source: Authors’ compilation.

**Fig. 5:**
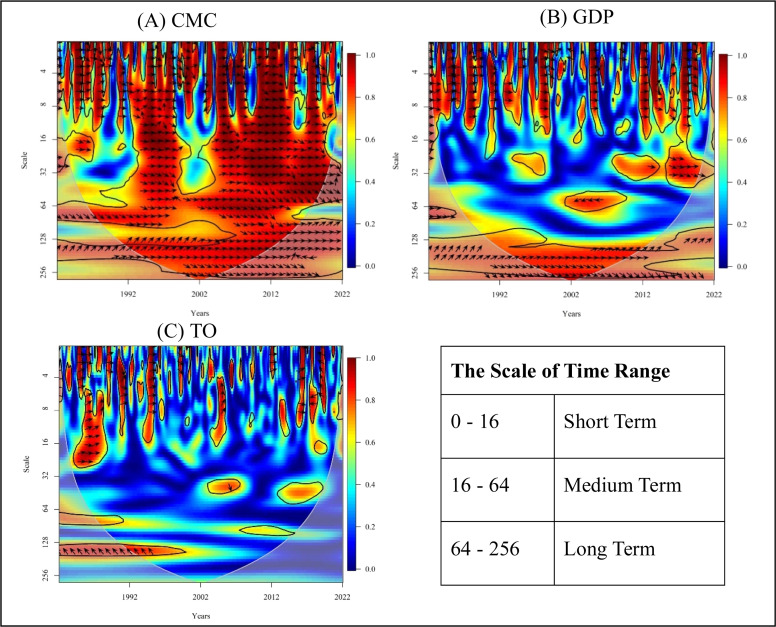
Wavelet coherence plot for the Asian region.

Source: Authors’ compilation using Posit RStudio (2025).

Wavelet coherence analysis was utilised across the Asian continent. The dependent variable was taken as CMP, and the independent variables considered were GDP and TO as presented in Fig. 5.

The correlation of the CMP and CMC is shown in Fig. 5 (A). The positive nature of coherence, with bidirectional causal flows between CMP and CMC, is observed throughout the short-term, medium-term, and long-term periods from 1993 to 2022 at a high frequency.

The relationship between the CMP and the GDP is illustrated in Fig. 5(B). The CMP and the GDP exhibit a two-way causality between the variables, with a positive coherence evident throughout the short-term period from 1993 to 2022. In the medium term, from 1993 to 2002, no causal flows between the variables are observed. In contrast, from 2002 to 2012, a negative, unidirectional causal relationship is evident, where CMP hurts GDP. Additionally, from 2013 to 2022, a positive one-way causality was observed from CMP to GDP in the medium term. Positive bidirectional causal flows can be observed between CMP and GDP from 1993 to 2012 in the long term; however, from 2012 to 2022, there is no significant long-term relationship between the variables.

The relationship between the CMP and the TO is illustrated in Fig. 5(C). A positive coherence with a bidirectional relationship between CMP and TO is observed from 1992 to 2002 in the short term, and then from 2003 to 2022. However, a negative coherence with unidirectional causality is observed, where CMP affects TO in the short term. There is no significant difference between the variables over the medium term. In the long term, from 1993 to 2002, a negative coherence with one-way causality is observed, where CMP affects TO; however, from 2003 to 2022, no significant correlation has been found.

The summary of the Wavelet coherence analysis results for the Asian continent is presented in Table 3 for a deeper understanding of the variables.

**Table 3:** Wavelet coherence analysis summary for the Asian region.

Note: A summary of wavelet coherence analysis between Chicken production (CMP) and each variable, including chicken meat consumption (CMC), Gross Domestic Product (GDP), and Trade openness (TO), for Asian countries, is illustrated in the tables. Results were obtained for short-term, medium-term, and long-term periods from 1993 to 2022. The nature of the coherence is indicated by the green up arrow sign, which represents positive coherence, the red down arrow sign represents negative coherence, and the black ‘X’ sign represents no coherence between the variables. The arrows represent the direction of causality. From the scale of frequency, red colour represents high frequency and yellow represents medium frequency.

Source: Authors’ compilation.

Wavelet coherence results have provided an overall perspective on the variables within the Asian continent. To obtain country-wise analysis for the variables, the following section concludes the Granger causality results.

### Granger causality analysis

Firstly, the DF test was used to test the stationarity of all variables, and the results are presented in Appendix 3. All variables in each country were stationary at the first difference (DCMP, DCMC, DGDP, DTO) while the Russian CMP variable was stationary at its second difference.

**Fig. 6:**
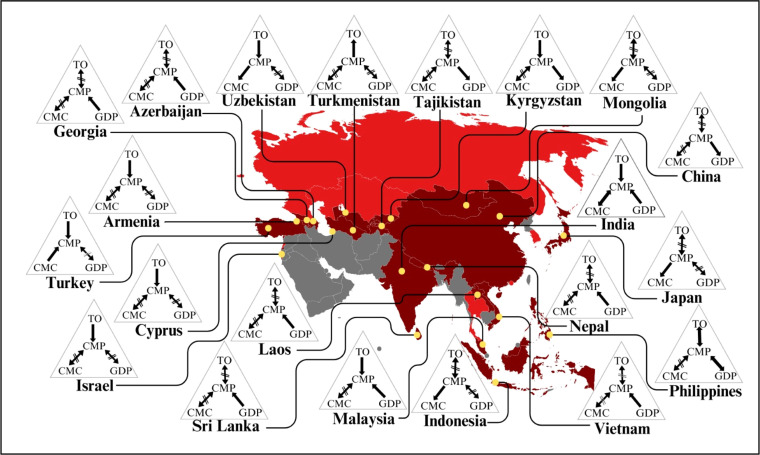
Granger causality results for Asian countries. Note: The triangle shapes in the figure represent the causal flows from chicken meat production (CMP) to chicken meat consumption (CMC), GDP, and trade openness (TO), respectively.

Source: Authors’ illustrations-based Granger analysis results.

Afterwards, use the VAR model to identify the optimal lag length using AIC, SBIC, and SQIC criteria. In the stability check, all eigenvalues lie within the unit root circle, confirming the stability of the model. Once all prerequisite tests are done, proceed with the Granger causality analysis, and the results are presented in Appendix 4. All these Granger causality analysis results were visualised in Fig. 6 to easily identify the direction of causality in each country in all variables.

The Granger results suggest a bidirectional causal relationship between chicken meat production and consumption in India. In Indonesia, Japan, Mongolia, and Uzbekistan, there is a unidirectional causal flow from chicken meat production to consumption. In contrast, Turkey exhibits a one-way causal relationship between chicken meat consumption and production. These findings are consistent with consumer demand theory, which suggests that chicken meat production is steady due to an increase in demand in the domestic markets.

Additionally, this situation aligns with past studies (Dagdemir et al., 2004). However, past studies in Japan (Ohtsu, 2025) suggest that the inefficiency of poultry farmers was the primary challenge in meeting the growing demand in the poultry industry. On the other hand, past studies indicate a clear association between chicken meat production and consumption in Japan (Imaizumi & Wallace, 2023).

Asian nations, such as China, Tajikistan, and Vietnam, exhibit one-way causality from chicken meat production to GDP, whereas Azerbaijan, India, Kyrgyzstan, Laos, Malaysia, Nepal, and Sri Lanka display a one-way causal flow from GDP to chicken meat production. Past studies suggest that India's economic growth has led to an increase in its poultry production and demand (Ali, 2015; Patil, 2025; United States Department of Agriculture, 2004) showed contradict causation like due to chicken production in Indian rural areas increases the national GDP by 1% with increasement of more than 4 million of employment opportunities..

In Sri Lanka, due to political instability, economic crisis, and the COVID-19 pandemic, poultry production has also significantly declined in recent years (Priyashantha and Vidanarachchi, 2024). This suggests the results were consistent with this observation, which indicates that increases in national income are clearly associated with poultry production growth in Sri Lanka.

Laos' growing livestock production enhances its national income (Millar and Photakoun, 2008; Vang et al., 2003) by expanding the trade market. Although in Kyrgyzstan, the development of poultry production is expected to increase revenue (Food and Agriculture Organisation, 2021). Vietnam also shows (Food and Agriculture Organisation, 2025a) a clear link between chicken meat production and the income level of households. These results suggest that the livestock industry plays a key role in reducing poverty (Agus et al., 2026; Pica-Ciamarra, 2005; Thieme & Pilling, 2008) in developing nations.

However, the Philippines shows a bidirectional casualty between chicken meat production, GDP, and trade openness. This aligns with past studies (Chang, 2007) by aligning with the results. Granger results in Turkmenistan indicate a relationship between unilateral casualties and chicken meat production, as well as trade openness. On the other hand, Armenia, Cyprus, Israel, Kyrgyzstan, Malaysia, Turkey, and Uzbekistan show a unidirectional relationship between trade openness and chicken meat production. These results support the international trade theory, which suggests that open markets enable countries to enhance their domestic chicken production for global markets.

Recent studies also suggest that Armenia has unilateral progression towards CMP (Silva et al., 2026) and the lower cost of importing frozen chicken than producing domestic chicken meat (Mkrtchyan et al., 2021). Therefore, most consumers tend to consume imported meat due to its lower cost. In India, due to non-tariff barriers, chicken meat exports face challenges. However, studies suggest that most African countries are open to importing Indian chicken meat. In recent years, India has experienced a 40.87% growth in poultry exports (Jagadeesh et al., 2024), suggesting a clear correlation between trade and chicken meat production, but did not find evidence that trade openness directly increases domestic chicken meat production. This highlights the novelty of the finding suggests that the trade and production nexus may vary across different economic contexts.

However, there were no statistically significant causal flows in any of the variables shown in Hong Kong, Kazakhstan, Lebanon, Macao, Russia, South Korea, and Thailand.

Post 1998, the bird flu crisis and a weak economy in Hong Kong affected chicken production, as well as the trade market (Harbert & Yuen, 2002; Thorburn & Yuen, 1998). Past studies have revealed that Russia is the fourth or fifth largest producer of broiler meat in the world and a growing nation in terms of chicken meat consumption. However, due to epizootic disease, the trade market decreased at the beginning of 2021 (Marinchenko, 2025; Rtishchev et al., 2023). In South Korea, consumers tend to prefer native Korean chicken over commercial broiler chicken due to its superior nutritional value, flavour, and texture (Jayasena et al., 2013; Jayasena et al., 2015). Thailand is a major producer, consumer, and exporter of chicken meat (United States Department of Agriculture, 2025a).

On the other hand, past studies revealed that increasing commercial broiler chicken meat production may enhance sustainability by reducing environmental impacts (Bungsrisawat et al., 2025). In Lebanon, the Syrian crisis, COVID-19, and ongoing political turmoil have led to an economic crisis. Due to a lack of agricultural infrastructure, high production costs, low incomes of rural farmers, and low consumer demand, the agrarian sector is facing issues in enhancing its production.

Additionally, due to the insufficient quality of production, the trade market faces barriers (Dal et al., 2021). These findings suggest that nations showing no causal progression between all variables should focus more on technology (Delgado et al., 1999; Zhang et al., 2017), innovations, and sustainable strategies to enhance the poultry industry within the Asian region.

Therefore, most past studies have explored the association of these variables separately in selected countries across different time periods within the Asian region, which can be attributed to the fluctuation between the retrieved results in this study.

All these Granger results were consisting with the Wavelet coherence analysis and this strength the study results were well described. To validate these Granger results, robustness checks are performed using different alternative lags and mentioned in Appendix 5. The most of robustness results align with the Granger causality results, thereby confirming the reliability of the Granger results in this study.

## Conclusion

In conclusion, this study examines the relationship between chicken meat production and three independent variables - chicken meat consumption, gross domestic product, and trade openness - for the Asian continent from 1993 to 2022. The majority of the Wavelet coherence analysis and Granger causality analysis results revealed bilateral causalities between chicken meat production with consumption, and GDP. Therefore, policy initiators should focus on using advanced technologies to help with the operational process and motivate domestic farmers to increase production. Additionally, the Wavelet coherence results were aligned with the Granger causality results, hence confirming the reliability of the retrieved results.

Finally, Policy implications for countries have been provided to enhance international trade and national income by promoting innovative and sustainable chicken meat production, while supporting the achievement of SDG goals. Further research using structural or experimental approach could further validate the causal mechanism of the present findings.

## Policy recommendations

Through a comprehensive analysis, the results provide empirical signals that may inform policy consideration. These policies aim to enhance chicken meat production in countries within the Asian region and promote the sustainability of the chicken industry, ultimately contributing to the United Nations' SDGs. These recommendations are derived from statistically observed dynamic relationships and are intended as evidence-informed guidelines rather than deterministic policy prescriptions.

The policies that correspond with the CMP and CMC, which have two-way casualties, such as those in India, should focus on enhancing production and consumption by investing in advanced technology that can help with the feeding process, production process, disease control, and improving distribution systems by deploying a network link between producers and retailers. Additionally, countries where CMP influences CMC, such as Indonesia, Japan, Mongolia, and Uzbekistan, should invest more in infrastructure and incentivise producers to achieve sustainable production. Then, countries such as Turkey, where CMC influences CMP, should shift the focus to product diversification to cater to the different preferences of consumers. Promote healthier options, such as chicken, to achieve consistent meat consumption. Analysing demand and consumption fluctuations and identifying trends will help reduce overproduction. These policy suggestions cater to SDG 2: Zero Hunger, SDG 3: Good Health and Well-being, SDG 8: Decent Work and Economic Growth, SDG 9: Industry, Innovation, and Infrastructure, and SDG 12: Responsible Consumption and Production.

The policies that correspond to CMP and GDP, with a bidirectional causality, such as the Philippines, should focus on growing the chicken meat sector by making public investments to improve infrastructure and give proper training to maximise production, since it highly contributes to the economy, and uses it to create opportunities to create jobs in the rural areas for poultry farming. Furthermore, countries such as China, Tajikistan, and Vietnam, where CMP has a significant impact on GDP, should focus on employing policies that involve the private sector in making investments and forming partnerships between the private and public sectors to enhance the production process and increase gross domestic product. This will enhance other sectors such as transportation, retail, and cold chain logistics, which will have a significant impact on the GDP. Azerbaijan, Georgia, India, Kyrgyzstan, Laos, Malaysia, Nepal, and Sri Lanka demonstrate that GDP affects CMPP; therefore, they should prioritise developing policies and regulations for poultry production to achieve consistent growth while reducing emissions. Through R&D, they can also provide environmentally friendly production methods. These efforts are aligned with the UN SDG 1: No Poverty, SDG 8: Decent Work and Economic Growth, SDG 9: Industry, Innovation, and Infrastructure, and SDG 17: Partnerships for the Goals.

The policies that correspond to CMP and TO suggest that countries with bidirectional trade, such as the Philippines, should focus on making trade agreements that will increase the competitiveness of local production and gain a higher share of the export market. Suppose there is one-way causality from CMP to TO, as in Turkmenistan. In that case, they should enhance the standards of certification for the export of poultry meat and promote production diversity to mitigate the risk of relying on a single marketplace. Improve the logistics facilities to export production. Suppose there is one-way causality from TO to CMP, as seen in Armenia, Cyprus, India, Israel, Kyrgyzstan, Malaysia, Türkiye, and Uzbekistan. In that case, they should focus on negotiating agreements for export expansion, which can also facilitate the expansion of chicken production. Additionally, adopting sustainable farming practices in poultry production can improve the quality, thereby promoting the export market. All the policies will cater to SDG 9: Industry, Innovation, and Infrastructure, SDG 12: Responsible Consumption and Production, and SDG 17: Partnerships for the Goals.

## Limitations and future research

This study provides valuable insights into the Asian continent, but it has several limitations. Only chicken meat was considered, and the focus was on the selected variables (gross domestic product, trade openness, chicken meat production, and chicken meat consumption) due to the data availability for the period. There are other variables, like land use and water use, that can be utilised. Furthermore, the Asian region comprises a total of 49 countries and five transcontinental countries; however, due to data unavailability, only a select few countries were included, resulting in the omission of others, which poses a limitation to the study. This study has utilised Wavelet coherence and Granger causality methodologies only.

Furthermore, this study provides an opportunity for future researchers to expand this study to other continents and include more countries to enhance the global impact of economic sustainability including employment generation, rural income distribution, and food security outcomes. Additionally, the study could be developed by including other meat types, extending the timeline, and employing more advanced methodologies to achieve more comprehensive results.

Further research could extend this framework using machine learning approaches, spatial estimators or multi-dimensional heterogeneous panel techniques to further explore non-linearities and regional clustering panels. Such an extension could complement rather than replace the frequency domain insight provided in this study. Despite its limitations, this study is hoped to make a significant contribution to the sustainability of the chicken industry and the agricultural sector.

## Statements and declarations

All authors certify that they have no affiliations with or involvement in any organization or entity with any financial interest or non-financial interest in the subject matter or materials discussed in this manuscript.

## Conflict of interest

The authors declare that they have no competing interests.

## Data availability statement

All data generated or analysed during this study are included in this published article and its N supplementary information files.

## Funding statement

This research did not receive any specific grant from funding agencies in the public, commercial, or not-for-profit sector

## Funding

The authors did not receive support from any organization for the submitted work.

## Clinical trial number

Not applicable

## Ethical approval

Not applicable. This study used publicly available secondary data, and no direct involvement of human participants, human tissue, or identifiable personal data was required.

## Consent to participate

Not Applicable

## Consent for publication

Not Applicable

## CRediT authorship contribution statement

**Yasodara Silva:** Writing – review & editing, Writing – original draft, Visualization, Validation, Software, Methodology, Formal analysis, Data curation, Conceptualization. **Himaya Susan:** Writing – review & editing, Writing – original draft, Visualization, Validation, Software, Methodology, Formal analysis, Data curation. **Nisal Perera:** Writing – review & editing, Writing – original draft, Visualization, Validation, Software, Methodology, Data curation. **Kalana Mendis:** Writing – review & editing, Writing – original draft, Visualization, Validation, Data curation. **Ruwan Jayathilaka:** Writing – review & editing, Writing – original draft, Visualization, Validation, Supervision, Resources, Project administration, Methodology, Investigation, Funding acquisition, Conceptualization. **Umesha Dabare:** Writing – review & editing, Writing – original draft, Validation, Project administration.

## Disclosures

The authors declare that they have no known competing financial interests or personal relationships that could have appeared to influence the work reported in this paper.

## Appendix Supplementary materials

Image, application 1

Image, application 2

Image, application 3

Image, application 4

Image, application 5
