# Supplementary material for: Dynamic linkages between chicken meat production, consumption, income and trade: Evidence from Wavelet coherence and Granger causality in Asia
Source: Poult Sci. 2026 Mar 6;105(6):106733. doi: 10.1016/j.psj.2026.106733 (PMC13018938; doi:10.1016/j.psj.2026.106733)
Supplement: Supplementary file 2 [file mmc2.docx]

**Appendix 2: Summary Descriptive Statistic of Variables**

| **Countries and variables** | | **Descriptive Statistics** | | | |
| --- | --- | --- | --- | --- | --- |
|  |  | **Mean** | **SD** | **Min** | **Max** |
| **Asian region** | CMP | 11.31 | 13.782 | 0.01 | 69.926 |
|  | CMC | 14.59 | 15.724 | 0.01 | 76.21 |
|  | GDP | 9694.21 | 14522.99 | 159.65 | 92815.59 |
|  | TO | 91.717 | 61.891 | 15.723 | 443 |
| **Armenia** | CMP | 2.456 | 1.301 | 1.04 | 5.259 |
|  | CMC | 10.327 | 5.444 | 1.336 | 19.62 |
|  | GDP | 2617.72 | 1855.905 | 390.55 | 7001.863 |
|  | TO | 77.112 | 14.004 | 54.542 | 112.429 |
| **Azerbaijan** | CMP | 6.916 | 4.183 | 2.087 | 13.748 |
|  | CMC | 8.106 | 4.182 | 2.767 | 16.06 |
|  | GDP | 3704.51 | 2996.372 | 177.99 | 8678.023 |
|  | TO | 86.315 | 16.727 | 55.349 | 133.49 |
| **China** | CMP | 8.685 | 2.087 | 4.299 | 11.506 |
|  | CMC | 11.093 | 3.284 | 4.788 | 17.69 |
|  | GDP | 4835.37 | 4259.426 | 418.2 | 13306.46 |
|  | TO | 43.443 | 9.780 | 32.424 | 64.479 |
| **Cyprus** | CMP | 29.368 | 7.475 | 19.324 | 40.22 |
|  | CMC | 27.02 | 2.166 | 23.761 | 30.33 |
|  | GDP | 18157.3 | 5484.938 | 8719.8 | 27045.32 |
|  | TO | 129.6 | 23.475 | 95 | 201 |
| **Georgia** | CMP | 4.092 | 1.635 | 1.956 | 7.244 |
|  | CMC | 11.168 | 5.479 | 1.957 | 19.32 |
|  | GDP | 2924.87 | 2012.382 | 565.47 | 7209.593 |
|  | TO | 87.513 | 24.202 | 45.697 | 166.903 |
| **Hong Kong** | CMP | 6.473 | 3.230 | 2.961 | 14.776 |
|  | CMC | 49.689 | 5.742 | 37.68 | 58.34 |
|  | GDP | 34703.4 | 9770.951 | 21717 | 51444.93 |
|  | TO | 331.133 | 73.126 | 220 | 443 |
| **India** | CMP | 1.886 | 1.014 | 0.718 | 3.813 |
|  | CMC | 1.693 | 0.902 | 0.656 | 3.49 |
|  | GDP | 1214.56 | 716.262 | 351.22 | 2559.901 |
|  | TO | 38.069 | 11.713 | 19.652 | 55.794 |
| **Indonesia** | CMP | 7.687 | 3.964 | 3.179 | 16.027 |
|  | CMC | 6.736 | 3.334 | 2.967 | 14.8 |
|  | GDP | 2509.53 | 1517.821 | 510.28 | 5145.763 |
|  | TO | 52.607 | 12.480 | 32.972 | 96.186 |
| **Israel** | CMP | 55.958 | 14.039 | 30.113 | 69.926 |
|  | CMC | 62.618 | 10.270 | 43.327 | 76.21 |
|  | GDP | 32942.8 | 12307.38 | 16904 | 60793.09 |
|  | TO | 64.301 | 7.668 | 51.151 | 79.608 |
| **Japan** | CMP | 13.228 | 3.754 | 9.877 | 19.628 |
|  | CMC | 17.494 | 3.174 | 13.736 | 27.09 |
|  | GDP | 40565.5 | 4213.339 | 34011 | 51285.82 |
|  | TO | 27.458 | 8.142 | 15.723 | 46.844 |
| **Kazakhstan** | CMP | 6.355 | 4.085 | 1.633 | 15.801 |
|  | CMC | 11.552 | 6.775 | 2.403 | 21.82 |
|  | GDP | 6739.01 | 4810.126 | 1186 | 15096.25 |
|  | TO | 76.954 | 14.619 | 53.05 | 105.7 |
| **Kyrgyzstan** | CMP | 1.314 | 0.486 | 0.673 | 2.982 |
|  | CMC | 4.016 | 2.625 | 0.689 | 10.73 |
|  | GDP | 907.917 | 501.444 | 291.36 | 1966.794 |
|  | TO | 104.469 | 23.457 | 71.824 | 146.106 |
| **Laos** | CMP | 4.059 | 1.301 | 2.313 | 6.347 |
|  | CMC | 3.686 | 1.450 | 1.915 | 6.45 |
|  | GDP | 1305.57 | 1001.02 | 290.74 | 2913.435 |
|  | TO | 72.605 | 13.666 | 52.084 | 99 |
| **Lebanon** | CMP | 23.356 | 5.044 | 16.25 | 31.133 |
|  | CMC | 20.174 | 2.516 | 14.486 | 23.86 |
|  | GDP | 6144.01 | 2319.64 | 2376.19 | 10101 |
|  | TO | 75.698 | 18.400 | 50.115 | 114.89 |
| **Macao** | CMP | 8.783 | 3.128 | 5.018 | 15.401 |
|  | CMC | 29.908 | 6.401 | 19.23 | 49.61 |
|  | GDP | 43749.1 | 27734.6 | 16145.1 | 92816 |
|  | TO | 130.667 | 15.475 | 112 | 168 |
| **Malaysia** | CMP | 42.067 | 7.516 | 31.997 | 57.521 |
|  | CMC | 41.383 | 8.675 | 32.092 | 56.69 |
|  | GDP | 8032.21 | 3167.99 | 3749.27 | 12606 |
|  | TO | 169.141 | 32.287 | 116.788 | 220.41 |
| **Mongolia** | CMP | 0.066 | 0.044 | 0.009 | 0.184 |
|  | CMC | 1.622 | 1.903 | 0.01 | 7.1 |
|  | GDP | 2503.93 | 1919.43 | 413.154 | 5595.3 |
|  | TO | 112.462 | 13.921 | 77.974 | 136.33 |
| **Nepal** | CMP | 1.825 | 2.413 | 0.502 | 9.759 |
|  | CMC | 1.637 | 2.150 | 0.434 | 8.7 |
|  | GDP | 650.699 | 433.607 | 205.53 | 1530.4 |
|  | TO | 47.855 | 6.416 | 36.297 | 64.036 |
| **Philippines** | CMP | 10.472 | 2.638 | 6.394 | 15.181 |
|  | CMC | 10.287 | 3.425 | 5.536 | 16.74 |
|  | GDP | 2262.12 | 1025.06 | 1084.64 | 3888 |
|  | TO | 69.2 | 11.367 | 50 | 88 |
| **Russia** | CMP | 17.686 | 11.654 | 4.407 | 38.812 |
|  | CMC | 20.874 | 8.549 | 6.647 | 34.23 |
|  | GDP | 8238.391 | 5287.068 | 1392.557 | 16981.79 |
|  | TO | 52.9 | 7.029 | 43 | 69 |
| **South Korea** | CMP | 13.361 | 3.930 | 8.784 | 20.919 |
|  | CMC | 14.393 | 4.508 | 8.567 | 22.56 |
|  | GDP | 22227.7 | 8744.93 | 8937.17 | 36239 |
|  | TO | 74.007 | 16.456 | 46.919 | 105.57 |
| **Sri Lanka** | CMP | 6.221 | 2.842 | 2.528 | 11.531 |
|  | CMC | 5.668 | 2.493 | 2.202 | 10.56 |
|  | GDP | 2443.77 | 1569.45 | 671.758 | 4778 |
|  | TO | 61.924 | 17.118 | 37.089 | 88.636 |
| **Tajikistan** | CMP | 0.55 | 0.862 | 0.017 | 3.575 |
|  | CMC | 1.876 | 1.556 | 0.019 | 4.22 |
|  | GDP | 652.249 | 388.385 | 159.646 | 1262 |
|  | TO | 101.361 | 40.950 | 49.938 | 181.59 |
| **Thailand** | CMP | 20.536 | 3.631 | 14.279 | 26.54 |
|  | CMC | 12.758 | 1.322 | 10.892 | 16.78 |
|  | GDP | 4546.65 | 2011.59 | 2011.19 | 7999 |
|  | TO | 116.793 | 18.104 | 77.746 | 140.44 |
| **Turkmenistan** | CMP | 2.919 | 1.277 | .423 | 4.612 |
|  | CMC | 3.997 | 2.468 | .388 | 11.19 |
|  | GDP | 3897.65 | 3017.98 | 618.836 | 9191.8 |
|  | TO | 85.569 | 37.880 | 33.058 | 170.26 |
| **Türkiye** | CMP | 18.834 | 8.413 | 5.496 | 31.288 |
|  | CMC | 14.755 | 5.066 | 7.079 | 21.44 |
|  | GDP | 8300.45 | 3776.9 | 2451.96 | 13708 |
|  | TO | 51.033 | 9.644 | 33 | 81 |
| **Uzbekistan** | CMP | 1.61 | 1.192 | 0.443 | 4.83 |
|  | CMC | 1.969 | 1.426 | 0.636 | 6.47 |
|  | GDP | 1519.31 | 980.310 | 425.295 | 3139.1 |
|  | TO | 60.456 | 19.869 | 29.192 | 114.03 |
| **Vietnam** | CMP | 6.297 | 3.504 | 1.937 | 14.765 |
|  | CMC | 8.349 | 4.800 | 2.34 | 19.62 |
|  | GDP | 1685.95 | 1378.73 | 217.37 | 4442.2 |
|  | TO | 128.614 | 31.010 | 66.212 | 186.68 |

Note: SD represent the Standard Deviation. CMP, CMC, GDP and TO represents Chicken Meat Production, Chicken Meat Consumption, Gross Domestic Product and Trade Openness respectively. Number of observations for all countries is equal to 30 (N = 30) and for Asian region is 810 (N = 810).
