# Supplementary material for: Dynamic linkages between chicken meat production, consumption, income and trade: Evidence from Wavelet coherence and Granger causality in Asia
Source: Poult Sci. 2026 Mar 6;105(6):106733. doi: 10.1016/j.psj.2026.106733 (PMC13018938; doi:10.1016/j.psj.2026.106733)
Supplement: Supplementary file 3 [file mmc3.docx]

**Appendix 3. The dicky-fuller unit root test results; classified by countries in Asian region**

| **Countries** | **CMP** | **DCMP** | | **CMC** | | **DCMC** | | **GDP** | | | **DGDP** | | **TO** | | **DTO** |
| --- | --- | --- | --- | --- | --- | --- | --- | --- | --- | --- | --- | --- | --- | --- | --- |
| Armenia | 0.069 | -6.895*** | | -1.207 | | -8.215*** | | 0.854 | | | -3.216** | | -2.351 | | -4.222*** |
| Azerbaijan | 0.866 | -5.197*** | | 0.421 | | -5.706*** | | -0.581 | | | -2.756* | | -4.58*** | |  |
| China | -2.571* |  | | -0.269 | | -4.607*** | | 2.432 | | | -4.601*** | | -1.191 | | -3.676** |
| Cyprus | -0.494 | -5.891*** | | -2.721* | |  | | -1.373 | | | -4.072*** | | 0.607 | | -3.287** |
| Georgia | -0.556 | -5.221*** | | -0.903 | | -6.107*** | | 0.718 | | | -2.890** | | -2.808* | |  |
| Hong Kong | -2.655* | -7.219*** | | -2.892** | | -5.750*** | | 0.091 | | | -4.673*** | | -1.206 | | -4.737*** |
| India | 3.879 | -3.158** | | 0.549 | | -7.578*** | | 1.041 | | | -5.751*** | | -1.323 | | -4.497*** |
| Indonesia | 0.852 | -3.024** | | 0.186 | | -6.212*** | | 0.491 | | | -3.979*** | | -2.513 | | -7.997*** |
| Israel | -1.875 | | -5.152*** | | -1.789 | | -4.940*** | | 1.414 | -5.205*** | | -1.577 | | -4.460*** | |
| Japan | 0.307 | | -4.529*** | | 2.164 | | -3.798*** | | -2.324 | -4.121*** | | -0.326 | | -4.238*** | |
| Kazakhstan | 1.922 | | -3.711*** | | 0.145 | | -4.884*** | | -0.613 | -3.674*** | | -1.571 | | -5.520*** | |
| Kyrgyzstan | -1.519 | | -3.199** | | -1.203 | | -5.334*** | | 0.703 | -3.886*** | | -1.427 | | -3.016** | |
| Laos | 0.502 | | -4.847*** | | 1.020 | | -4.710*** | | -0.378 | 0.313 | | -1.726 | | -4.241*** | |
| Lebanon | -1.879 | | -5.092*** | | -2.139 | | -5.071*** | | -1.499 | -2.812* | | -1.323 | | -3.382** | |
| Macao | -2.122 | | -6.880*** | | 1.189 | | -3.417** | | -1.307 | -4.781*** | | -1.040 | | -4.260*** | |
| Malaysia | -0.690 | | -3.789*** | | -0.276 | | -4.624*** | | -0.541 | -4.980*** | | -0.780 | | -3.951*** | |
| Mongolia | -1.602 | | -6.170*** | | 3.930 | | -2.198 | | 0.111 | -3.697** | | -2.699* | |  | |
| Nepal | -0.666 | | -6.000*** | | -0.584 | | -5.756*** | | 1.779 | -3.840*** | | -1.957 | | -4.933*** | |
| Philippines | -0.800 | | -4.085*** | | -0.037 | | -4.796*** | | 0.332 | -4.801*** | | -1.895 | | -3.448** | |
| Russia | -0.168 | | -4.727***^D2^ | | -0.168 | | -4.518*** | | -0.532 | -3.803*** | | -2.811* | |  | |
| South Korea | -0.030 | | -5.050*** | | -0.006 | | -5.401*** | | -0.836 | -4.898*** | | -1.533 | | -4.636*** | |
| Sri Lanka | 0.187 | | -5.424*** | | 0.192 | | -6.206*** | | -0.859 | -2.795* | | -0.796 | | -4.328*** | |
| Tajikistan | -1.305 | | -5.381*** | | -1.334 | | -5.660*** | | -0.009 | -3.263** | | -1.537 | | -5.208*** | |
| Thailand | -0.927 | | -6.425*** | | -2.451 | | -5.939*** | | -0.170 | -3.736*** | | -2.264 | | -5.116*** | |
| Turkmenistan | -0.629 | | -4.242*** | | -0.588 | | -6.482*** | | 0.669 | -3.769*** | | -1.334 | | -4.218*** | |
| Türkiye | -0.041 | | -6.048*** | | -0.594 | | -5.661*** | | -1.058 | -4.781*** | | -0.471 | | -4.822*** | |
| Uzbekistan | 1.965 | | -3.709*** | | 1.664 | | -3.432** | | -0.113 | -2.777* | | -3.069** | |  | |
| Vietnam | 2.250 | | -3.964*** | | -0.979 | | -4.763*** | | 3.477 | -2.623* | | -1.145 | | -5.232*** | |

Note: *** Significance at 1%, ** Significance at 5%, * Significance at 10%. Note: *** Significance at 1%, ** Significance at 5%, * Significance at 10%.
