# Supplementary material for: Dynamic linkages between chicken meat production, consumption, income and trade: Evidence from Wavelet coherence and Granger causality in Asia
Source: Poult Sci. 2026 Mar 6;105(6):106733. doi: 10.1016/j.psj.2026.106733 (PMC13018938; doi:10.1016/j.psj.2026.106733)
Supplement: Supplementary file 4 [file mmc4.docx]

**Appendix 4. Summary of cross-country granger causality test results**

| **Country** | **DCMP**  **DCMC** | **DCMC**  **DCMP** | **DCMP**  **DGDP** | **DGDP**  **DCMP** | **DCMP**  **DTO** | **DTO**  **DCMP** |
| --- | --- | --- | --- | --- | --- | --- |
| Armenia | 0.828 | 0.568 | 0.53248 | 0.08583 | 1.4779 | 4.7911* |
| Azerbaijan | 0.33258 | 0.99493 | 1.6795 | 9.147*** | 1.1217 | 2.4058 |
| China | 0.10055 | 1.1831 | 14.786*** | 0.48868 | 0.49781 | 1.7539 |
| Cyprus | 0.12706 | 2.6469 | 0.33146 | 1.8306 | 2.382 | 2.8388* |
| Georgia | 3.8116 | 0.17235 | 4.2357 | 6.4908** | 0.0795 | 0.0345 |
| Hong Kong | 1.5201 | 0.63564 | 2.5028 | 2.4075 | 0.59632 | 3.138 |
| India | 9.0389*** | 7.0883*** | 0.20482 | 8.8656*** | 0.90498 | 15.693*** |
| Indonesia | 7.8134*** | 0.75736 | 1.1541 | 1.0698 | 0.34088 | 0.08181 |
| Israel | 2.846 | 1.7582 | 1.6304 | 4.5108 | 0.77011 | 4.7069* |
| Japan | 6.6791** | 0.0176 | 1.6169 | 1.6905 | 1.6604 | 1.0267 |
| Kazakhstan | 1.0021 | 4.1095 | 0.01878 | 6.2277 | 1.8165 | 0.03823 |
| Kyrgyzstan | 1.8151 | 2.0754 | 0.24971 | 4.6466* | 1.1433 | 9.5307*** |
| Laos | 0.40768 | 0.25182 | 0.24971 | 4.6466* | 2.5407 | 2.0389 |
| Lebanon | 0.13833 | 0.32503 | 3.5746 | 0.08013 | 0.66962 | 1.7191 |
| Macao | 2.0874 | 0.23915 | 0.10511 | 0.5369 | 1.4714 | 0.16822 |
| Malaysia | 0.72243 | 0.89799 | 1.3669 | 17.927*** | 0.52979 | 4.7969* |
| Mongolia | 3.2637* | 2.2744 | 1.9018 | 3.1701 | 1.3201 | 0.41195 |
| Nepal | 1.6329 | 1.6193 | 1.7565 | 5.0083** | 2.0299 | 0.86003 |
| Philippines | 2.4473 | 2.1904 | 7.8771*** | 5.422** | 3.8803** | 4.0575** |
| Russia | 0.02423 | 0.0077 | 1.4701 | 2.4737 | 1.3164 | 0.00273 |
| South Korea | 1.4126 | 0.94608 | 2.1916 | 1.7189 | 1.096 | 0.24033 |
| Sri Lanka | 1.4126 | 0. 94608 | 1.3262 | 5.6089* | 0.00822 | 2.1335 |
| Tajikistan | 0.40243 | 4.6031 | 5.05018* | 0.72058 | 0.03749 | 0.18246 |
| Thailand | 0.56238 | 0.03548 | 1.13 | 2.1377 | 1.2179 | 1.7364 |
| Turkmenistan | 0.95997 | 1.7554 | 0.2678 | 1.9845 | 7.9704* | 1.611 |
| Türkiye | 0.13642 | 5.3361* | 1.3062 | 2.2792 | 0.87729 | 12.773*** |
| Uzbekistan | 10.929*** | 1.0986 | 0.01011 | 1.3904 | 0.00074 | 4.1987** |
| Vietnam | 2.4651 | 0.18534 | 7.7083*** | 1.8758 | 1.86 | 0.49784 |

Note: The symbols *, **, and *** denote significance at the 10%, 5%, and 1% levels, respectively. CMP, CMC, GDP, and TO refer to chicken meat production, chicken meat consumption, gross domestic product, and trade openness, respectively. DCMP, DCMC, DGDP, and DTO indicate the first-differenced forms of each variable.
