# Supplementary material for: Dynamic linkages between chicken meat production, consumption, income and trade: Evidence from Wavelet coherence and Granger causality in Asia
Source: Poult Sci. 2026 Mar 6;105(6):106733. doi: 10.1016/j.psj.2026.106733 (PMC13018938; doi:10.1016/j.psj.2026.106733)
Supplement: Supplementary file 5 [file mmc5.docx]

**Appendix 5. Robustness check; classified by Asian countries**

1. **Armenia**

**Alternative lag 01**

| Lag Length Used |  | Alternative Lag 01 |
| --- | --- | --- |
| 1 | DCMP DCMC | 0.3024 |
|  | DCMC DCMP | 0.8096 |
| 1 | DCMP DGDP | 0.68521 |
|  | DGDP DCMP | 0.00236 |
| 1 | DCMP TO | 0.45214 |
|  | TO DCMP | 1.267 |

**Alternative lag 02**

| Lag Length Used |  | Alternative Lag 02 |
| --- | --- | --- |
| 2 | DCMP DCMC | 0.00037 |
|  | DCMC DCMP | 0.00909 |
| 2 | DCMP DGDP | 3.4 |
|  | DGDP DCMP | 0.00415 |
| 2 | DCMP TO | 0.49316 |
|  | TO DCMP | 1.4744 |

1. **Azerbaijan**

**Alternative lag 01**

| Lag Length Used |  | Alternative Lag 01 |
| --- | --- | --- |
| 1 | DCMP DCMC | 0.47216 |
|  | DCMC DCMP | 0.97664 |
| 0 | DCMP DGDP | 6.8038** |
|  | DGDP DCMP | 8.9753** |
| 0 | DCMP TO | 0.06321 |
|  | TO DCMP | 1.297 |

**Alternative lag 02**

| Lag Length Used |  | Alternative Lag 02 |
| --- | --- | --- |
| 2 | DCMP DCMC | 0.03193 |
|  | DCMC DCMP | 0.06348 |
| 2 | DCMP DGDP | 0.16609 |
|  | DGDP DCMP | 0.00032 |
| 2 | DCMP TO | 1.1012 |
|  | TO DCMP | 0.69307 |

1. **China**

**Alternative lag 01**

| Lag Length Used |  | Alternative Lag 01 |
| --- | --- | --- |
| 0 | CMP DCMC | 0.16019 |
|  | DCMC CMP | 0.07979 |
| 0 | CMP DGDP | 20.331*** |
|  | DGDP CMP | 1.9183 |
| 0 | CMP TO | 1.7126 |
|  | TO CMP | 2.7459 |

**Alternative lag 02**

| Lag Length Used |  | Alternative Lag 02 |
| --- | --- | --- |
| 2 | CMP DCMC | 0.07398 |
|  | DCMC CMP | 0.75617 |
| 2 | CMP DGDP | 8.6855*** |
|  | DGDP CMP | 1.4189 |
| 2 | CMP TO | 0.92021 |
|  | TO CMP | 1.6664 |

1. **Cyprus**

**Alternative lag 01**

| Lag Length Used |  | Alternative Lag 01 |
| --- | --- | --- |
| 0 | DCMP CMC | 0.38191 |
|  | CMC DCMP | 2.745 |
| 1 | DCMP DGDP | 0.09305 |
|  | DGDP DCMP | 0.12698 |
| 0 | DCMP TO | 13.898*** |
|  | TO DCMP | 8.1337** |

**Alternative lag 02**

| Lag Length Used |  | Alternative Lag 02 |
| --- | --- | --- |
| 2 | DCMP CMC | 0.02515 |
|  | CMC DCMP | 1.1281 |
| 2 | DCMP DGDP | 0.06125 |
|  | DGDP DCMP | 1.4166 |
| 2 | DCMP TO | 14.486*** |
|  | TO DCMP | 7.3722*** |

1. **Georgia**

**Alternative lag 01**

| Lag Length Used |  | Alternative Lag 01 |
| --- | --- | --- |
| 1 | DCMP DCMC | 0.81123 |
|  | DCMC DCMP | 0.11589 |
| 1 | DCMP DGDP | 0.85129 |
|  | DGDP DCMP | 4.9076** |
| 0 | DCMP TO | 0.7185 |
|  | TO DCMP | 0.09607 |

**Alternative lag 02**

| Lag Length Used |  | Alternative Lag 02 |
| --- | --- | --- |
| 2 | DCMP DCMC | 1.8405 |
|  | DCMC DCMP | 0.01575 |
| 2 | DCMP DGDP | 0.39816 |
|  | DGDP DCMP | 0.0001 |
| 2 | DCMP TO | 0.27716 |
|  | TO DCMP | 0.02032 |

1. **Hong Kong**

**Alternative lag 01**

| Lag Length Used |  | Alternative Lag 01 |
| --- | --- | --- |
| 1 | DCMP DCMC | 3.7459* |
|  | DCMC DCMP | 0.49723 |
| 1 | DCMP DGDP | 0.86215 |
|  | DGDP DCMP | 0.00067 |
| 1 | DCMP TO | 0.12458 |
|  | TO DCMP | 2.1225 |

**Alternative lag 02**

| Lag Length Used |  | Alternative Lag 02 |
| --- | --- | --- |
| 2 | DCMP DCMC | 0.19389 |
|  | DCMC DCMP | 0.90591 |
| 2 | DCMP DGDP | 0.79919 |
|  | DGDP DCMP | 1.9966 |
| 2 | DCMP TO | 0.541 |
|  | TO DCMP | 0.00232 |

1. **India**

**Alternative lag 01**

| Lag Length Used |  | Alternative Lag 01 |
| --- | --- | --- |
| 0 | DCMP DCMC | 8.586** |
|  | DCMC DCMP | 8.2928** |
| 0 | DCMP DGDP | 2.4838 |
|  | DGDP DCMP | 18.176*** |
| 1 | DCMP TO | 0.60862 |
|  | TO DCMP | 3.3177* |

**Alternative lag 02**

| Lag Length Used |  | Alternative Lag 02 |
| --- | --- | --- |
| 2 | DCMP DCMC | 0.0777 |
|  | DCMC DCMP | 0.11119 |
| 2 | DCMP DGDP | 1.4336 |
|  | DGDP DCMP | 0.01011 |
| 2 | DCMP TO | 0.95314 |
|  | TO DCMP | 5.7357** |

1. **Indonesia**

**Alternative lag 01**

| Lag Length Used |  | Alternative Lag 01 |
| --- | --- | --- |
| 1 | DCMP DCMC | 7.8888*** |
|  | DCMC DCMP | 3.9564** |
| 1 | DCMP DGDP | 0.64194 |
|  | DGDP DCMP | 0.17735 |
| 1 | DCMP DTO | 0.00073 |
|  | DTO DCMP | 0.08952 |

**Alternative lag 02**

| Lag Length Used |  | Alternative Lag 02 |
| --- | --- | --- |
| 3 | DCMP DCMC | 0.01977 |
|  | DCMC DCMP | 2.0081 |
| 3 | DCMP DGDP | 0.35685 |
|  | DGDP DCMP | 0.58224 |
| 3 | DCMP DTO | 0.00143 |
|  | DTO DCMP | 1.265 |

1. **Israel**

**Alternative lag 01**

| Lag Length Used |  | Alternative Lag 01 |
| --- | --- | --- |
| 1 | DCMP DCMC | 0.7764 |
|  | DCMC DCMP | 1.1301 |
| 1 | DCMP DGDP | 0.09958 |
|  | DGDP DCMP | 0.05154 |
| 1 | DCMP DTO | 0.75658 |
|  | DTO DCMP | 2.7609* |

**Alternative lag 02**

| Lag Length Used |  | Alternative Lag 02 |
| --- | --- | --- |
| 2 | DCMP DCMC | 1.2173 |
|  | DCMC DCMP | 0.3985 |
| 2 | DCMP DGDP | 1.4169 |
|  | DGDP DCMP | 4.2425** |
| 2 | DCMP DTO | 0.48016 |
|  | DTO DCMP | 1.4874 |

1. **Japan**

**Alternative lag 01**

| Lag Length Used |  | Alternative Lag 01 |
| --- | --- | --- |
| 1 | DCMP DCMC | 6.6718*** |
|  | DCMC DCMP | 0.04281 |
| 1 | DCMP DGDP | 0.69761 |
|  | DGDP DCMP | 0.22471 |
| 1 | DCMP DTO | 1.1506 |
|  | DTO DCMP | 0.09764 |

**Alternative lag 02**

| Lag Length Used |  | Alternative Lag 02 |
| --- | --- | --- |
| 2 | DCMP DCMC | 0.32678 |
|  | DCMC DCMP | 0.0083 |
| 2 | DCMP DGDP | 1.6445 |
|  | DGDP DCMP | 1.7683 |
| 2 | DCMP DTO | 0.19374 |
|  | DTO DCMP | 0.93259 |

1. **Kazakhstan**

**Alternative lag 01**

| Lag Length Used |  | Alternative Lag 01 |
| --- | --- | --- |
| 1 | DCMP DCMC | 1.2986 |
|  | DCMC DCMP | 1.4364 |
| 2 | DCMP DGDP | 1.635 |
|  | DGDP DCMP | 0.04242 |
| 1 | DCMP DTO | 1.3923 |
|  | DTO DCMP | 0.08427 |

**Alternative lag 02**

| Lag Length Used |  | Alternative Lag 02 |
| --- | --- | --- |
| 2 | DCMP DCMC | 0.68077 |
|  | DCMC DCMP | 0.07505 |
| 4 | DCMP DGDP | 0.04774 |
|  | DGDP DCMP | 0.04801 |
| 2 | DCMP DTO | 0.116 |
|  | DTO DCMP | 0.00992 |

1. **Kyrgyzstan**

**Alternative lag 01**

| Lag Length Used |  | Alternative Lag 01 |
| --- | --- | --- |
| 1 | DCMP DCMC | 0.75353 |
|  | DCMC DCMP | 1.768 |
| 1 | DCMP DGDP | 0.0385 |
|  | DGDP DCMP | 0.00203 |
| 1 | DCMP DTO | 0.63922 |
|  | DTO DCMP | 0.31678 |

**Alternative lag 02**

| Lag Length Used |  | Alternative Lag 02 |
| --- | --- | --- |
| 2 | DCMP DCMC | 0.01281 |
|  | DCMC DCMP | 0.19571 |
| 2 | DCMP DGDP | 0.22054 |
|  | DGDP DCMP | 4.3346** |
| 2 | DCMP DTO | 0.57299 |
|  | DTO DCMP | 7.3736*** |

1. **Laos**

**Alternative lag 01**

| Lag Length Used |  | Alternative Lag 01 |
| --- | --- | --- |
| 1 | DCMP DCMC | 0.38098 |
|  | DCMC DCMP | 7.9 |
| 0 | DCMP DGDP | 0.19663 |
|  | DGDP DCMP | 0.15872 |
| 1 | DCMP DTO | 1.2326 |
|  | DTO DCMP | 2.0609 |

**Alternative lag 02**

| Lag Length Used |  | Alternative Lag 02 |
| --- | --- | --- |
| 2 | DCMP DCMC | 4.56 |
|  | DCMC DCMP | 0.25183 |
| 2 | DCMP DGDP | 0.40716 |
|  | DGDP DCMP | 0.0512 |
| 2 | DCMP DTO | 1.5816 |
|  | DTO DCMP | 0.41366 |

1. **Lebanon**

**Alternative lag 01**

| Lag Length Used |  | Alternative Lag 01 |
| --- | --- | --- |
| 1 | DCMP DCMC | 0.3212 |
|  | DCMC DCMP | 0.13812 |
| 1 | DCMP DGDP | 0.87732 |
|  | DGDP DCMP | 0.11999 |
| 1 | DCMP DTO | 0.52024 |
|  | DTO DCMP | 0.32301 |

**Alternative lag 02**

| Lag Length Used |  | Alternative Lag 02 |
| --- | --- | --- |
| 2 | DCMP DCMC | 0.04928 |
|  | DCMC DCMP | 0.26183 |
| 2 | DCMP DGDP | 0.52859 |
|  | DGDP DCMP | 0.03295 |
| 2 | DCMP DTO | 0.25227 |
|  | DTO DCMP | 1.4572 |

1. **Macao**

**Alternative lag 01**

| Lag Length Used |  | Alternative Lag 01 |
| --- | --- | --- |
| 1 | DCMP DCMC | 2.0431 |
|  | DCMC DCMP | 0.01395 |
| 1 | DCMP DGDP | 0.01252 |
|  | DGDP DCMP | 0.1977 |
| 1 | DCMP DTO | 1.8885 |
|  | DTO DCMP | 0.00062 |

**Alternative lag 02**

| Lag Length Used |  | Alternative Lag 02 |
| --- | --- | --- |
| 2 | DCMP DCMC | 0.00076 |
|  | DCMC DCMP | 0.03304 |
| 2 | DCMP DGDP | 0.09603 |
|  | DGDP DCMP | 0.18283 |
| 2 | DCMP DTO | 0.05145 |
|  | DTO DCMP | 0.03962 |

1. **Malaysia**

**Alternative lag 01**

| Lag Length Used |  | Alternative Lag 01 |
| --- | --- | --- |
| 1 | DCMP DCMC | 0.20145 |
|  | DCMC DCMP | 0.54302 |
| 1 | DCMP DGDP | 0.10243 |
|  | DGDP DCMP | 0.2494 |
| 1 | DCMP DTO | 0.03759 |
|  | DTO DCMP | 4.3774** |

**Alternative lag 02**

| Lag Length Used |  | Alternative Lag 02 |
| --- | --- | --- |
| 2 | DCMP DCMC | 0.33539 |
|  | DCMC DCMP | 0.37978 |
| 2 | DCMP DGDP | 0.8985 |
|  | DGDP DCMP | 14.651*** |
| 2 | DCMP DTO | 0.05478 |
|  | DTO DCMP | 0.68664 |

1. **Mongolia**

**Alternative lag 01**

| Lag Length Used |  | Alternative Lag 01 |
| --- | --- | --- |
| 3 | DCMP DCMC | 5.1037** |
|  | DCMC DCMP | 12.326*** |
| 1 | DCMP DGDP | 1.3773 |
|  | DGDP DCMP | 1.6324 |
| 1 | DCMP TO | 3.6121* |
|  | TO DCMP | 1.52 |

**Alternative lag 02**

| Lag Length Used |  | Alternative Lag 02 |
| --- | --- | --- |
| 5 | DCMP DCMC | 0.66276 |
|  | DCMC DCMP | 0.04226 |
| 2 | DCMP DGDP | 0.3892 |
|  | DGDP DCMP | 1.8299 |
| 3 | DCMP TO | 6.1094** |
|  | TO DCMP | 0.15011 |

1. **Nepal**

**Alternative lag 01**

| Lag Length Used |  | Alternative Lag 01 |
| --- | --- | --- |
| 2 | DCMP DCMC | 1.5157 |
|  | DCMC DCMP | 1.4011 |
| 1 | DCMP DGDP | 1.1178 |
|  | DGDP DCMP | 0.03151 |
| 1 | DCMP DTO | 0 .147 |
|  | DTO DCMP | 0.00179 |

**Alternative lag 02**

| Lag Length Used |  | Alternative Lag 02 |
| --- | --- | --- |
| 4 | DCMP DCMC | 0.0034 |
|  | DCMC DCMP | 0.00534 |
| 3 | DCMP DGDP | 0.46599 |
|  | DGDP DCMP | 6.4617** |
| 2 | DCMP DTO | 1.2354 |
|  | DTO DCMP | 0.79144 |

1. **Philippines**

**Alternative lag 01**

| Lag Length Used |  | Alternative Lag 01 |
| --- | --- | --- |
| 0 | DCMP DCMC | 6.2173** |
|  | DCMC DCMP | 2.1312 |
| 1 | DCMP DGDP | 1.2753 |
|  | DGDP DCMP | 1.6264 |
| 2 | DCMP DTO | 1.2091 |
|  | DTO DCMP | 0.02886 |

**Alternative lag 02**

| Lag Length Used |  | Alternative Lag 02 |
| --- | --- | --- |
| 2 | DCMP DCMC | 0.12268 |
|  | DCMC DCMP | 0.30741 |
| 3 | DCMP DGDP | 0.32192 |
|  | DGDP DCMP | 0.02851 |
| 4 | DCMP DTO | 1.3692 |
|  | DTO DCMP | 0.57454 |

1. **Russia**

**Alternative lag 01**

| Lag Length Used |  | Alternative Lag 01 |
| --- | --- | --- |
| 3 | DCMP DCMC | 0.77029 |
|  | DCMC DCMP | 0.02251 |
| 3 | DCMP DGDP | 0.60075 |
|  | DGDP DCMP | 0.46202 |
| 0 | DCMP TO | 5.1314 |
|  | TO DCMP | 0.42549 |

**Alternative lag 02**

| Lag Length Used |  | Alternative Lag 02 |
| --- | --- | --- |
| 5 | DCMP DCMC | 0.25038 |
|  | DCMC DCMP | 1.1336 |
| 5 | DCMP DGDP | 0.07653 |
|  | DGDP DCMP | 0.01834 |
| 2 | DCMP TO | 2.4926 |
|  | TO DCMP | 0.16193 |

1. **South Korea**

**Alternative lag 01**

| Lag Length Used |  | Alternative Lag 01 |
| --- | --- | --- |
| 1 | DCMP DCMC | 2.7294* |
|  | DCMC DCMP | 1.0257 |
| 1 | DCMP DGDP | 2.2858 |
|  | DGDP DCMP | 0.45914 |
| 1 | DCMP DTO | 0.18116 |
|  | DTO DCMP | 0.29605 |

**Alternative lag 02**

| Lag Length Used |  | Alternative Lag 02 |
| --- | --- | --- |
| 2 | DCMP DCMC | 0.00083 |
|  | DCMC DCMP | 0.96128 |
| 2 | DCMP DGDP | 0.10327 |
|  | DGDP DCMP | 1.5784 |
| 2 | DCMP DTO | 1.0004 |
|  | DTO DCMP | 0.00015 |

1. **Sri Lanka**

**Alternative lag 01**

| Lag Length Used |  | Alternative Lag 01 |
| --- | --- | --- |
| 2 | DCMP DCMC | 0.26927 |
|  | DCMC DCMP | 2.918* |
| 1 | DCMP DGDP | 0.76871 |
|  | DGDP DCMP | 0.05061 |
| 1 | DCMP DTO | 0.07693 |
|  | DTO DCMP | 1.9691 |

**Alternative lag 02**

| Lag Length Used |  | Alternative Lag 02 |
| --- | --- | --- |
| 4 | DCMP DCMC | 0.21084 |
|  | DCMC DCMP | 0.31502 |
| 2 | DCMP DGDP | 0.29557 |
|  | DGDP DCMP | 4.9233** |
| 2 | DCMP DTO | 0.01538 |
|  | DTO DCMP | 0.16184 |

1. **Tajikistan**

**Alternative lag 01**

| Lag Length Used |  | Alternative Lag 01 |
| --- | --- | --- |
| 1 | DCMP DCMC | 0.10213 |
|  | DCMC DCMP | 2.3542 |
| 1 | DCMP DGDP | 0.5642 |
|  | DGDP DCMP | 0.51126 |
| 1 | DCMP DTO | 0.03364 |
|  | DTO DCMP | 0.21589 |

**Alternative lag 02**

| Lag Length Used |  | Alternative Lag 02 |
| --- | --- | --- |
| 2 | DCMP DCMC | 0.08122 |
|  | DCMC DCMP | 0.84818 |
| 2 | DCMP DGDP | 4.0935** |
|  | DGDP DCMP | 0.01629 |
| 2 | DCMP DTO | 0.03178 |
|  | DTO DCMP | 0.02268 |

1. **Thailand**

**Alternative lag 01**

| Lag Length Used |  | Alternative Lag 01 |
| --- | --- | --- |
| 1 | DCMP DCMC | 0.22119 |
|  | DCMC DCMP | 0.01181 |
| 1 | DCMP DGDP | 0.0269 |
|  | DGDP DCMP | 0.00087 |
| 1 | DCMP DTO | 1.4296 |
|  | DTO DCMP | 0.32034 |

**Alternative lag 02**

| Lag Length Used |  | Alternative Lag 02 |
| --- | --- | --- |
| 2 | DCMP DCMC | 0.46841 |
|  | DCMC DCMP | 0.00809 |
| 2 | DCMP DGDP | 0.91197 |
|  | DGDP DCMP | 1.8506 |
| 2 | DCMP DTO | 0.80534 |
|  | DTO DCMP | 0.81325 |

1. **Turkmenistan**

**Alternative lag 01**

| Lag Length Used |  | Alternative Lag 01 |
| --- | --- | --- |
| 1 | DCMP DCMC | 0.99425 |
|  | DCMC DCMP | 0.07648 |
| 1 | DCMP DGDP | 0.26413 |
|  | DGDP DCMP | 3.3472* |
| 1 | DCMP DTO | 0.15681 |
|  | DTO DCMP | 2.1823 |

**Alternative lag 02**

| Lag Length Used |  | Alternative Lag 02 |
| --- | --- | --- |
| 2 | DCMP DCMC | 0.27364 |
|  | DCMC DCMP | 0.82857 |
| 2 | DCMP DGDP | 0.10009 |
|  | DGDP DCMP | 0.27685 |
| 2 | DCMP DTO | 7.058*** |
|  | DTO DCMP | 0.22376 |

1. **Turkey**

**Alternative lag 01**

| Lag Length Used |  | Alternative Lag 01 |
| --- | --- | --- |
| 1 | CMP DCMC | 0.1474 |
|  | DCMC CMP | 3.9681** |
| 1 | CMP DGDP | 0.91835 |
|  | DGDP CMP | 0.51035 |
| 0 | CMP TO | 4.3791 |
|  | TO CMP | 12.344*** |

**Alternative lag 02**

| Lag Length Used |  | Alternative Lag 02 |
| --- | --- | --- |
| 2 | CMP DCMC | 0.00136 |
|  | DCMC CMP | 0.43555 |
| 2 | CMP DGDP | 0.03241 |
|  | DGDP CMP | 1.2487 |
| 2 | CMP TO | 0.35454 |
|  | TO CMP | 22.16*** |

1. **Uzbekistan**

**Alternative lag 01**

| Lag Length Used |  | Alternative Lag 01 |
| --- | --- | --- |
| 2 | DCMP DCMC | 24.427*** |
|  | DCMC DCMP | 1.5152 |
| 0 | DCMP DGDP | 0.1337 |
|  | DGDP DCMP | 5.4518* |
| 1 | DCMP TO | 0.93691 |
|  | TO DCMP | 3.6123* |

**Alternative lag 02**

| Lag Length Used |  | Alternative Lag 02 |
| --- | --- | --- |
| 4 | DCMP DCMC | 4.006** |
|  | DCMC DCMP | 0.23114 |
| 2 | DCMP DGDP | 0.04582 |
|  | DGDP DCMP | 0.18075 |
| 3 | DCMP TO | 0.30069 |
|  | TO DCMP | 0.15542 |

1. **Vietnam**

**Alternative lag 01**

| Lag Length Used |  | Alternative Lag 01 |
| --- | --- | --- |
| 1 | DCMP DCMC | 0.83788 |
|  | DCMC DCMP | 0.18913 |
| 1 | DCMP DGDP | 1.655 |
|  | DGDP DCMP | 3.3728 |
| 1 | DCMP DTO | 1.1218 |
|  | DTO DCMP | 0.29685 |

**Alternative lag 02**

| Lag Length Used |  | Alternative Lag 02 |
| --- | --- | --- |
| 2 | DCMP DCMC | 0.29202 |
|  | DCMC DCMP | 0.08469 |
| 3 | DCMP DGDP | 4.166** |
|  | DGDP DCMP | 4.2286** |
| 2 | DCMP DTO | 0.05013 |
|  | DTO DCMP | 0.26845 |
